# Supplementary material for: Splice-Junction-Based Mapping of Alternative Isoforms in the Human Proteome
Source: Cell Rep. Author manuscript; Available in PMC 2020 Jan 15. (PMC6961840; doi:10.1016/j.celrep.2019.11.026)
Supplement: 2 [file NIHMS1546469-supplement-2.zip › DF1/PXD010154/Thyroid/Thyroid_47_EVI5_ESYSTTETLEK.pdf]

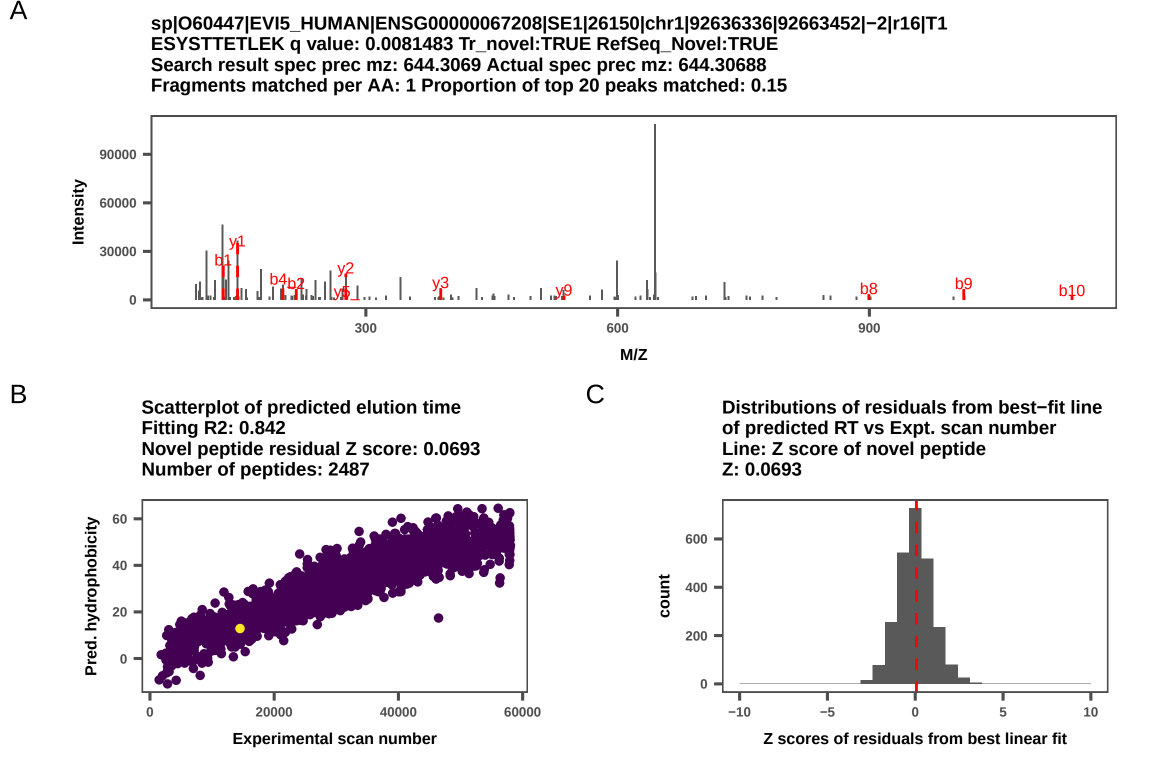
A
sp|O60447|EVI5_HUMAN|ENSG00000067208|SE1|26150|chr1|92636336|92663452|−2|r16|T1
ESYSTTETLEK q value: 0.0081483 Tr_novel:TRUE RefSeq_Novel:TRUE
Search result spec prec mz: 644.3069 Actual spec prec mz: 644.30688
Fragments matched per AA: 1 Proportion of top 20 peaks matched: 0.15
Intensity
90000
60000
30000
0
y1
b1
b4
b2
y2
y5
y3
y9
b8
b9
b10
300
600
900
M/Z
B
Scatterplot of predicted elution time
Fitting R2: 0.842
Novel peptide residual Z score: 0.0693
Number of peptides: 2487
Pred. hydrophobicity
60
40
20
0
0
20000
40000
60000
Experimental scan number
C
Distributions of residuals from best−fit line
of predicted RT vs Expt. scan number
Line: Z score of novel peptide
Z: 0.0693
count
600
400
200
0
−10
−5
0
5
10
Z scores of residuals from best linear fit
